# Titanium Implant Impairment and Surrounding Muscle Cell Death Following High-Salt Diet: An *In Vivo* Study

**DOI:** 10.1371/journal.pone.0146873

**Published:** 2016-01-13

**Authors:** Mathieu Lecocq, Marie-Solenne Felix, Jean-Marc Linares, Julien Chaves-Jacob, Patrick Decherchi, Erick Dousset

**Affiliations:** 1 Aix-Marseille Université, CNRS, Institut des Sciences du Mouvement: Etienne-Jules MAREY (UMR 7287), Equipe « Plasticité des Systèmes Nerveux et Musculaire » (PSNM), Parc Scientifique et Technologique de Luminy, Faculté des Sciences du Sport de Marseille, CC910, 163, avenue de Luminy, 13288, Marseille Cedex 09, France; 2 Aix-Marseille Université, CNRS, Institut des Sciences du Mouvement: Etienne-Jules MAREY (UMR 7287), Equipe « Conception Bio-Inspirée » (CBI), IUT d'Aix-Marseille, 413, avenue Gaston Berger, 13625, Aix-en–Provence Cedex, France; University of Akron, UNITED STATES

## Abstract

**Aim of the study:**

High-salt consumption has been widely described as a risk factor for cardiovascular, renal and bone functions. In the present study, the extent to which high-salt diet could influence Ti6Al4V implant surface characteristic, its adhesion to rat tibial crest, and could modify muscle cell viability of two surrounding muscles, was investigated in vivo. These parameters have also been assessed following a NMES (neuro-myoelectrostimulation) program similar to that currently used in human care following arthroplasty.

**Results:**

After a three-week diet, a harmful effect on titanium implant surface and muscle cell viability was noted. This is probably due to salt corrosive effect on metal and then release of toxic substance around biologic tissue. Moreover, if the use of NMES with high-salt diet induced muscles damages, the latter were higher when implant was added. Unexpectedly, higher implant-to-bone adhesion was found for implanted animals receiving salt supplementation.

**Conclusion:**

Our in vivo study highlights the potential dangerous effect of high-salt diet in arthroplasty based on titanium prosthesis. This effect appears to be more important when high-salt diet is combined with NMES.

## Introduction

Health institutes are warning the population about danger from high-salt diet which is considered to be a potent source of many disorders including cardiovascular diseases [1]. In industrialized population, average of sodium chloride (NaCl) uptake excels twice as much as the maximal recommended intake of 85 mmol sodium (Na^+^) per day (nearly 5g NaCl/day) [2,3]. Since the agricultural revolution, humans’ overconsumption of NaCl has reversed the K^+^/Na^+^ ratio from 10 K^+^ per 1 Na^+^ to more than 1 K^+^ per 3 Na^+^ [4]. Such high-salt diet could result in bone degradation and/or osteoporosis [5,6]. Bone loss was attributed to low-grade metabolic acidosis induced by an increase of NaCl intake that tends to rise with age [7–9]. Moreover, several authors have demonstrated high calcium (Ca^2+^) depletion in patients with high-salt diet [6,9,10]. Beside, according to Sarkis et al. [11], bioavailability of Ca^2+^ seems to be dependent of Na^+^ intake.

Considering the effects of high-salt diet on bones and given the high prevalence of arthroplasty prescribed every year for elderly people [12], the role of high-salt intake should be taken into account as a potential risk factor for implant loosening. Indeed, it was reported that implant loosening is mainly attributed to osteolysis [13]. More recently, Frings-Meuthen et al. reported after a high-salt diet, compared to a low-salt diet, a higher CTX rate (a bone resorption markers) and a 74% increase in urinary Ca^2+^ excretion in 24 hours [14]. In patients who remained bedridden during 14 days, inactivity-induced muscle loss was amplified when they received high-salt diet compared to that receiving low-salt diet (respectively, 70g of muscle loss versus 25g) [14]. On the other hand, high-salt diet could modify electrochemical balance of prosthesis surrounding tissues and emphasized the corrosion stress occurring on implant surface resulting in wear debris propagation. Indeed, saline solutions have already been described as a risk factor of pitting corrosion [15–17]. Thus, in patient who had undergone arthroplasty, high-salt diet could represent a potential risk of muscle weakness, osteoporosis, high corrosion level, wear debris product, and finally prosthesis loosening.

Prosthesis will be submitted to mechanical and chemical stress, generating wear debris dissemination [18–20]. Regardless to materials properties, the mechanical effect includes contact pressure, sliding velocity, lubrication while chemical effects originates from metal oxidation, dissolution and adsorption due to biological fluids [21]. After exposure of a Cobalt Chromium Molybdenum (CoCrMo) implant to a simulated physiological solution, Hedberg and Wallinder, concluded that precipitation of metal-ionic complexes is highly dependent of metal type, environment chemistry and time of exposure [22]. Furthermore, it was reported that corrosion process of metallic alloys, currently used in orthopedics and dental setting, originated from immersion of metal in rich-electrolytes biological environment [19]. Indeed, without any treatment, biological tissues are already known to exert a corrosive effect on metallic compound that increase risk of wear debris dissemination [23,24]. As a result, the main issue for arthroplasty with metal compound is to keep a low level of electrochemical stress to delay the implant wear and debris releasing.

Electrochemical reactions between the corroding metallic surface and the electrolytes can result in large currents induced by generated ions and electrons flow [19]. These currents could be amplified by electrical stimulation, used in post-arthroplasty human care to prevent post-surgical muscle weakness. Gittens et al. have already highlighted the risk of uncontrolled conduction of electrical currents through biological tissues with the use of neuro-myoelectrostimulation (NMES) [19]. It was also noted a 1000-fold higher release of chromium ions from CoCrMo alloy when a potential of 0.7 V was applied during 2h in PBS solution [22]. Recently, we demonstrated adverse effects of NMES on a Ti6Al4V implant fixed on rat tibial crest and muscle damages both on stimulated muscle and surrounding muscle suggesting an electrical propagation through the implant to the nearby tissue [22].

In the present *in vivo* study, we aimed to test the extent to which high-salt diet could affect implant-to-bone attachment and viability of surrounding muscle cells. More precisely, high-salt diet influences were measured on attachment of a Ti6Al4V implant placed on a tibial crest and on the surrounding muscles, namely the *flexor digitorum* (FD) and the *tibialis anterior* (TA). We also evaluated those influences after the application of a NMES program currently used for total knee arthroplasty rehabilitation. We hypothesized that high-salt diet could reduce the attachment of the implant on bone and increase damages in surrounding muscles. These high-salt diet consequences should be worst when a NMES program is applied.

## Materials and Methods

### 1. Models

Forty five adult male Sprague Dawley rats (12 weeks old), weighing 400 g (Centre d’Elevage Roger JANVIER^®^, Le Genest Saint Isle, France), were housed in smooth-bottomed, plastic cages at 22°C with a 12h light/dark cycle. Food (Safe^®^, Augy, France) and water were available *ad libitum*. An acclimation period of one week was allowed before the initiation of the experiment. All animals were weighed before each experimental step.

In order to model the implant, 3 rats were sacrificed and the left hind paw tibial bones were collected.

Rats were randomly assigned to seven experimental groups: 1) Control group (n = 6) which received no treatment; 2) Es group (n = 6) in which animals were solely submitted to two weeks of NMES sessions; 3) NaCl group (n = 6) submitted to high-salt diet for three weeks (no surgery was performed); 4) NaCl-Es group (n = 6) in which animals received 3 weeks of high-salt diet and were submitted to 2 weeks of NMES sessions one week following diet beginning; 5) Ti group (n = 6) in which animals received a Ti6Al4V alloy implant on the tibial crest; 6) Ti-NaCl group (n = 6) submitted to high-salt diet after implantation of the Ti6Al4V alloy; 7) Ti-NaCl-Es group (n = 6) in which animals received high-salt diet and were submitted to 2 weeks of NMES sessions one week following implantation of the Ti6Al4V alloy.

### 2. Ethical approval

Anesthesia and surgical procedures were performed according to the French law on animal care guidelines. The Animal Care Committees of *Aix-Marseille Université* (AMU) and *Centre National de la Recherche Scientifique* (CNRS) approved our protocols. Individuals conducting the research were listed in the authorized personnel section of the animal research protocol or added to a previously approved protocol (License A 13 01306). Furthermore, experiments were performed following the recommendations provided in the *Guide for Care and Use of Laboratory Animals* (U.S. Department of Health and Human Services, National Institutes of Health) and in accordance with the European Community’s council directive of 24 November 1986 (86/609/ EEC). No clinical sign of pain or unpleasant sensation (i.e. screech, prostration, hyperactivity, anorexia) or paw-eating behavior was observed throughout the study.

### 3. Implant design

**Fig 1:**
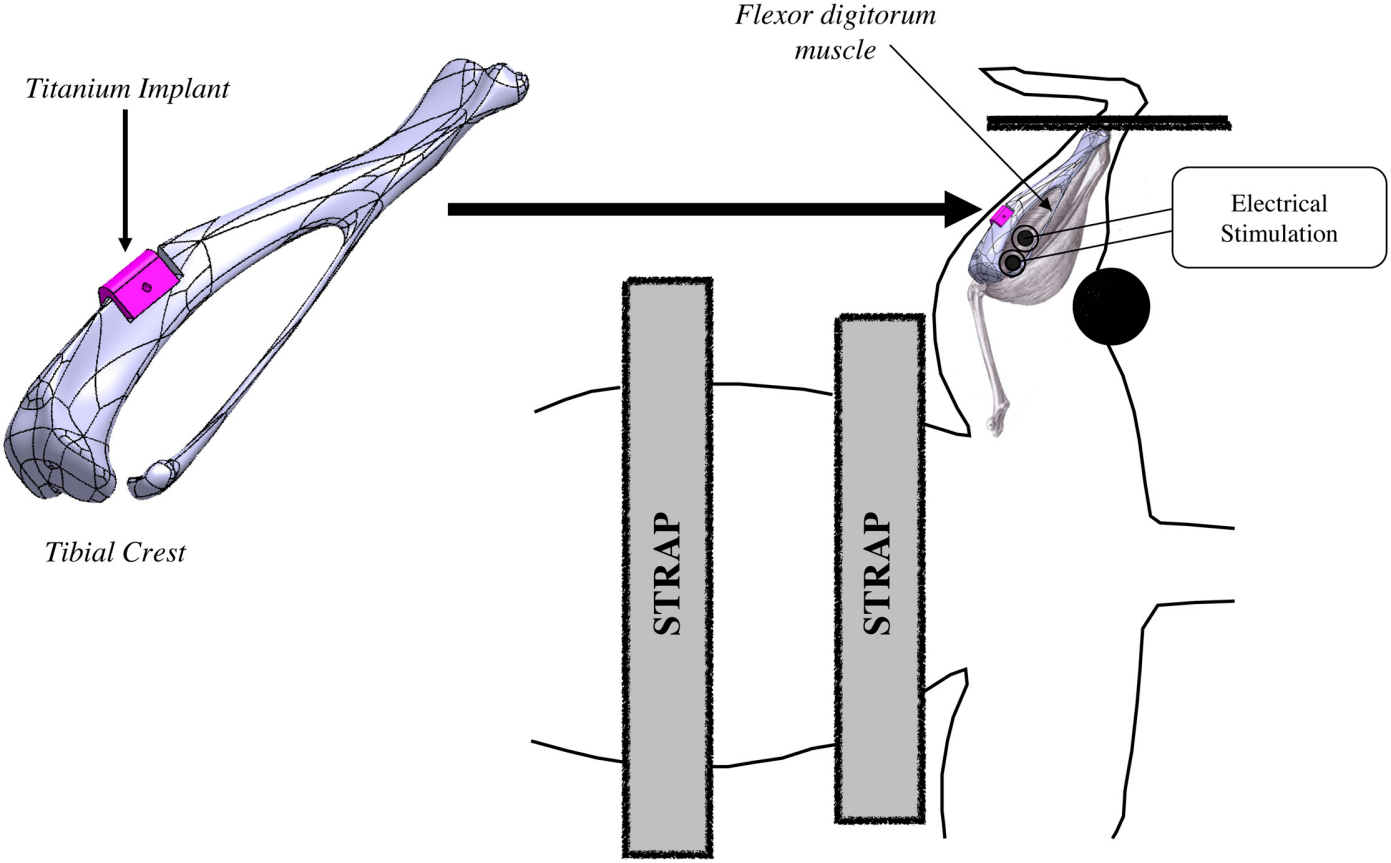
Design of the implant and NEMS device. Rat is positioned in supination. Straps located on thorax and abdomen avoid any movement interference. Foot is firmly held and stimulation electrodes are positioned on the skin right above the first 1/3 of FD muscle. Implant location is represented from digitalized implant and tibial bone made using a Computer Assisted Design system.

According to a previous study [25], we used an implant designed to fit a part of rat tibial crest without risking surrounding muscles injury during all the protocol. The implant size (4x3x3.5 mm) was chosen so that the classical surgery procedure (surgical pliers, without binocular microscope) could be used for implantation. The geometry of designed implant was standard for all experiments (Fig 1) and made from 3D scanner on tibia originating from rats previously sacrificed.

Implants were machined from Ti6Al4V material by a 5-axes micro-milling machine (US 20, Deckel Maho Gildemaster, Leonberg, Germany). The programming process for machining each piece was performed by an ISO standard program generated by the CATIA V5 system. Implants were numbered to facilitate identification and to determine the level of degradation at the end of the experiment. For the sake of validity, all implants were identical and had a triangular prism shape with a width of 3.5 mm, a length of 4 mm (largest base in contact with bone surface) and a height of 3 mm (Fig 1). After machining, implants received only a cleaning treatment without more particular surface treatment. A hole of 0.5 mm was machined at the middle of the implant to achieve the implant-to-bone adhesion test. One of the two lateral surfaces exposed to the muscle tissue was accurately measured (Micromesure 2, STIL SA, Aix-en-Provence, France) before implantation and after animal sacrifice to assess its deterioration. Comparison between these two acquired surfaces was achieved by a roughness software (SurfaceMap Software, Digital Surf, Besançon, France).

### 4. Surgical protocol

Rats from Ti, Ti-NaCl and Ti-NaCl-Es groups were anesthetized with an intraperitoneal injection of chloral hydrate (0.05 g/ml; 1 ml/100 g; Sigma Life Science, Saint-Louis, USA). The left hind paw was shaved and a 1.5 cm skin incision was made along the tibial bone. Muscles in contact with the tibial bone were carefully separated from the bone in order to avoid muscle damage. A portion of the bone, similar to the volume of the implant, was removed from the tibial crest using a micro-milling/grinder machine (Dremel 300 series multitool, Bosch^®^, Mount Prospect, USA). As in orthopedic surgery, the implant was anchored to the tibial bone with acrylic cement (CEMFIX 1 Teknimed S.A.S, L’Union, France). Cement was positioned on the surface of the implanted area. Then, the implant was fixed and cement was added on bone-implant interface adhesion. This method allows free contact between a large part of implant surface and surrounding tissues. After a drying delay, muscles were sutured at two points on either side of the implanted area. Finally, the skin was sutured and animals received local anesthetic (Lidocaine T7394c, Sigma-Aldrich, Saint-Louis, USA) subcutaneously around the implantation site to minimize pain.

### 5. High-salt diet

According to Safe^®^ Company which provided food for used rats, we have determined that NaCl level in the basic food ranged between 0.25 and 0.27% of food weight. Since most high-salt diets that were used in the scientific literature were composed with levels up to 8% NaCl [26], this value was chosen as reference. In accordance with Oloyo et al. food pellets usually given to rats were mixed with water to form a smooth paste, then salt (7.75% of the total food weight) was added to reach the desired salt concentration of 8% and pellets were reconstituted before being dried for 24 hours [27].

### 6. NMES program

Animals from Es, NaCl-Es and Ti-NaCl-Es groups were submitted to NMES program five days a week for two weeks with a program commonly used in clinical therapy for faster mobility recovery. Ti-NaCl-Es group was kept at rest for one week after surgical implantation for healing and recovery, and then submitted to the same NMES program. According to our previous protocol [25], in order to achieve stress-free sessions, rats were slightly anesthetized (right hind paw, contralateral to the studied hind paw) with a binary intramuscular mixture of 0.65 ml of ketamine (Ketamine 1000, Virbac, Carros, France) and 0.25 ml of largactil (Thorazine chlorpromazine, 0.1 ml per 100 g, Laboratory Aventis, Paris, France). Animals were placed in supine position and immobilized by slightly tensed straps located on thorax and abdomen (Fig 1). Ankle was firmly held with an angle of 90° to avoid movements and friction stimulations. Electrodes (ECG Electrodes universal, Medical Chart Control, Brie Comte Robert, France) were located on the skin and positioned strictly above FD muscle according to anatomical table. The correct electrodes position was insured by the fingers flexion induced by stimulation. Moreover, as FD arises from the tibia below popliteus, and from the head of the fibula, this muscle was not in contact with the implant avoiding any friction stress exerted on the implant. FD muscle was stimulated using a 75 Hz frequency and a 6.25 s duration separated by 20 s periods of active recovery (3 Hz stimulation) during around 20 min (40 stimulations). The choice of this program was based on literature indicating that high frequency stimulation applied to the quadriceps muscle after total knee arthroplasty allowed better strength and activation recoveries than lower frequencies. Stimulation intensity was determined from the motor threshold (MT). Then, intensity was increased by 0.1 x MT at each session from 1.1 x MT (during the first session) to 2 x MT (during the last session).

### 7. Muscle and bone sampling

At the end of the NMES session, animals from all groups were deeply anesthetized with chloral hydrate (0.05 g/ml, i.p.). FD and TA muscles were removed. The latter muscle was selected to assess eventual increase of muscle damage related to current propagation from FD to TA through the metallic implant. Sampling was performed 90 min after the last stimulation in stimulated groups. The implanted hind paw was incised from knee to ankle and FD and TA muscles were separated from surrounding tissue and carefully collected after section of the proximal and distal tendons. Immediately after collection, muscles were frozen in isopentane (2-méthylbuthane, Sigma-Aldrich^®^, Saint-Louis, USA) and stored at—80°C for further immunohistochemistry analysis.

Animals were then sacrificed by cervical dislocation. Implanted rats tibial bones were cut at both extremities (at least 4 mm on either side of the implant) using a micro-circular saw (Dremel 300 series multitool, Bosch^®^, Mount Prospect, USA), removed, and stored at -20°C before implant-to-bone adhesion measurement and analysis of the implant surface.

### 8. Implant-to-bone adhesion

**Fig 2:**
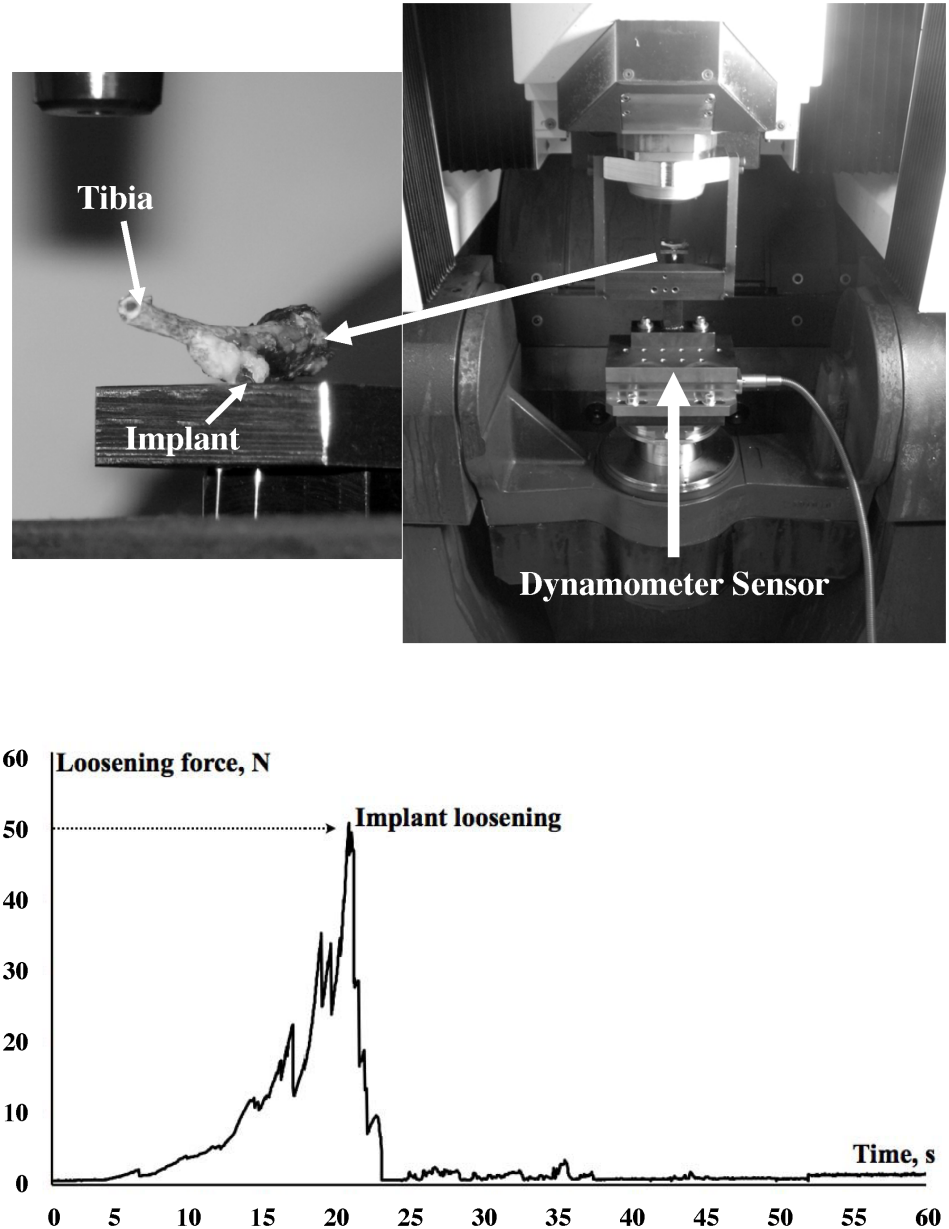
Tensile test. A homemade device is used to measure the adhesion load between the implant and the bone. Briefly, tibial bone is placed, implant downwards, on a flat metal bracket containing a hole allowing the implant to be loaded by a tension device. Highly resistant wire is passed through the implant hole then connected to the load system. The mobile part of the testing device is slowly displaced to stretch the wire until the implant loosened. Graphic in lower part indicates the time-curve of force recorded and the maximum load value obtained just before the breakout of the implant marked by a thin arrow.

Sectioned tibial bones were placed, implants downward, on a flat metal bracket containing a hole allowing the implant to be loaded by a tension device [25]. Briefly, highly resistant wire was passed through the implant hole then connected to the load system. The use of a wire permits the alignment of the implant during the tensile test. The mobile part of the testing device was slowly displaced to stretch the wire until the implant loosened. The applied displacement was controlled by numerical axis with controlled speed displacement (0.02 m/mn). During this experiment, the value of the force was recorded using a 10 Hz frequency dynamometer sensor with 0.01 N of resolution (Kistler^®^, Les Ulis, France). The maximum load value was obtained just before the breakout of the implant. It was recorded by acquisition software (Kistler^®^, Les Ulis, France). This value reflected the maximum tensile force required to loosen the implant (Fig 2).

### 9. Implant surface assessment

Before and after implantation, the plane surface of each implant was measured on an optical coordinate measuring machine (Micromesure 2, STIL SA, Aix-en-Provence, France), which analyzes the surface roughness with a resolution of 10 nm. Surface scanning was performed by point acquisition every 4 μm on the two plane directions of the surface. The two sets of points were next treated by roughness software (Mountain Map Universal Digital Surf, Besançon, France). Each measured set of points was processed in several stages: 1) best fit of the theoretical plane; 2) suppression of the best-fitted plane; 3) removal of the outlier points. Then, these two treated sets of implant surface points were used to calculate the altitude differences between the two surfaces, before and after implantation. From this difference, the range between the higher and the lower surface point (in microns) was chosen to estimate the implant impairment.

### 10. Muscle cell death analysis

FD and TA muscles were cut, on their entire length, into 50 μm-thick longitudinal slices using a cryostat (Leica Microsystems^®^, Wetzlar, Germany), collected on glass slides (Superfrost Plus Slat Thermo Scientific, Waltham, USA), rehydrated in a phosphate buffer solution (PBS), then blocked with PBS containing 10% normal donkey serum (NDS) and 0.2% Triton X-100. Approximately, 400 sections per muscle were then incubated overnight in the same blocking solution with a rabbit anti-activated caspase 3 (1/1000, Cell Signaling Technology^®^, Beverly, USA). Following several washes in PBS, sections were incubated for 2h in PBS containing 5% NDS with Alexa Fluor^®^ 488 donkey anti-rabbit antibody (1/400, Invitrogen^®^, Carlsbad, USA). Activated caspase 3 positive cells were quantified in muscles using an epifluorescence microscope (Leica Microsystems^®^, Wetzlar, Germany) to determine the apoptosis rate as the average number of dead cells per area of 0.3 cm^2^.

### 11. Statistical analysis

Data were expressed as the mean ± SEM (Standard Error Mean). The statistical treatment was performed using the R Instat software (GraphPad Software^®^, La Jolla, USA). Prior to any statistical test, normal distribution was checked. According to this result, analysis of variance (One-way ANOVA—group effect) was performed to compare implant-to-bone adhesion, surface analysis and muscle cell death rate. Significant effects were considered when p<0.05 and a *post-hoc* Bonferroni test was therefore applied. In order to measure the amount of surface degradation of each implant, a non-parametric Wilcoxon test (one-tailed) was performed.

## Results

### 1. Behavioral observations of animals

Throughout the protocol, drinking and food-pellet intake remained unchanged and no weight loss occurred for all the rats. Furthermore, no sign of pain, prostration, hyperactivity, immobility or aggressive behavior was observed for all animals.

### 2. Implant-to-bone adhesion

**Fig 3:**
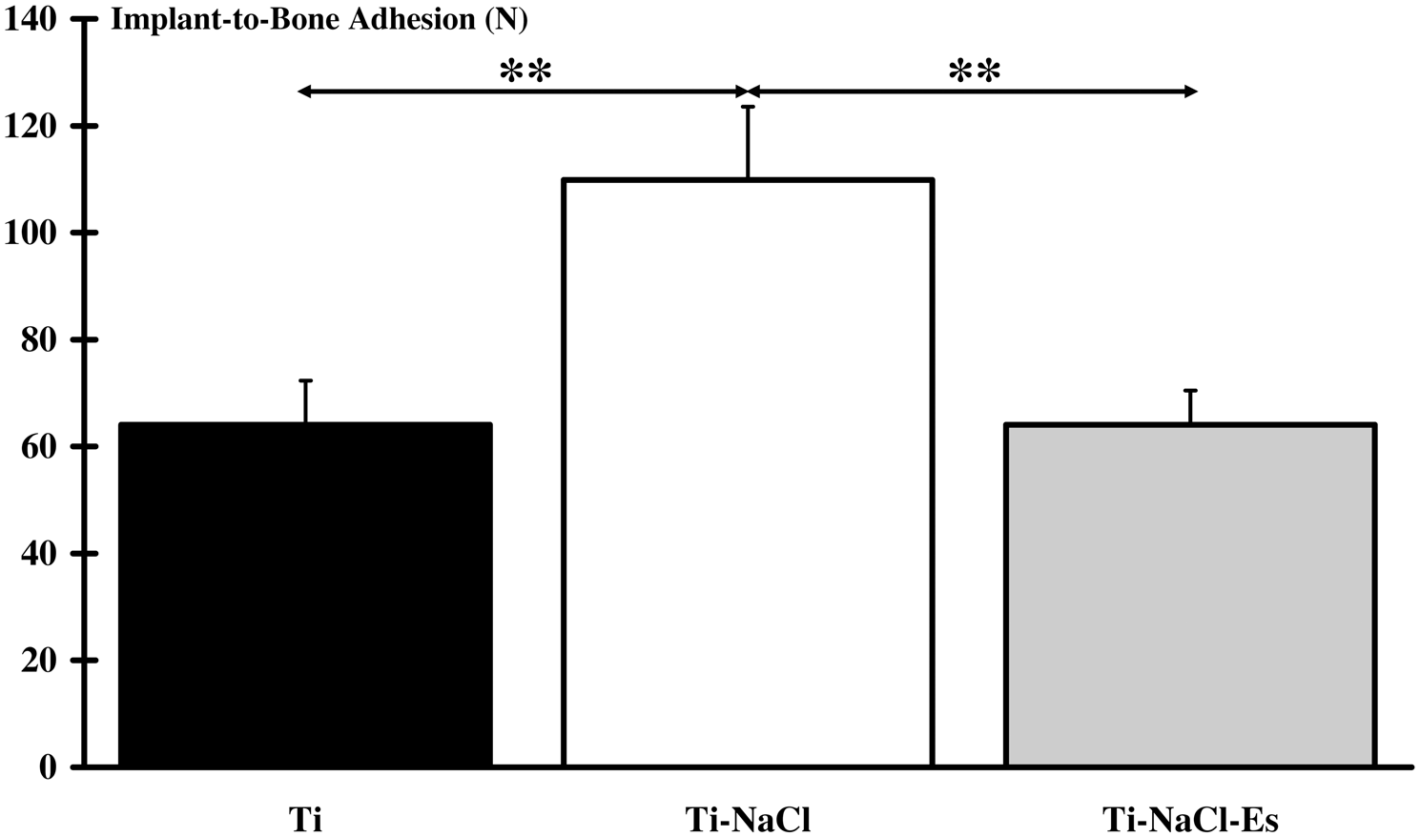
Implant-to-bone adhesion. Measurement of the maximum load value indicates that adhesion of the implants to the tibial crest bone is significantly (**: p<0.01) higher when implanted animals are submitted to high-salt diet (Ti-NaCl) compared to only implanted animals (Ti) or animals simultaneously submitted to high-salt diet and NEMS program (Ti-NaCl-Es).

Results of implant-to-bone adhesion are presented in Fig 3. Ti-NaCl group exhibited a higher adhesion (111.03±11.61 N, p<0.01) compared to the two other implanted groups (64.39±8.81 N for Ti and 64.72±4.37 N for Ti-NaCl-Es). No significant differences were found between Ti and Ti-NaCl-Es groups (S1 Fig).

### 3. Implant surface impairment

**Fig 4:**
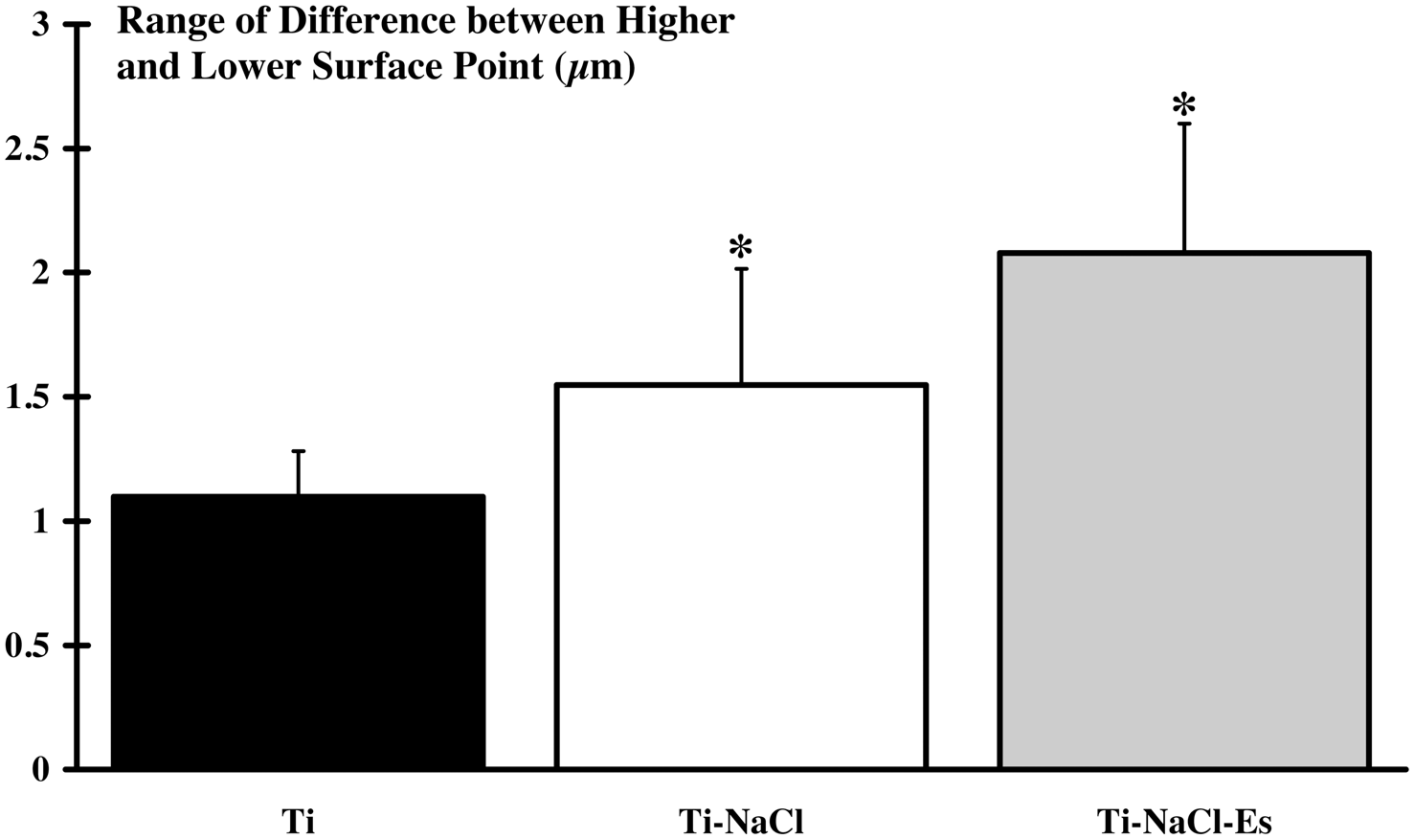
Surface degradation of the implant. The surface of the implant is scanned before and 3 weeks after implantation and the range between higher and lower surface point is measured. Results indicate that the Ti-NaCl (implanted and submitted to high-salt diet) and Ti-NaCl-Es (implanted and simultaneously submitted to high-salt diet and NEMS program) groups present a significant higher (*: p<0.05) degradation of implant compared to Ti (non submitted to high-salt diet and NEMS program) group.

The range of differences between the higher and the lower surface point was compared before and three weeks after implantation (Fig 4). Intergroup comparison did not reveal difference between the three implanted groups. However, surface degradation was significantly higher only for Ti-NaCl (1.55±0.44 μm; p<0.05) and Ti-NaCl-Es (2.09±0.49 μm; p<0.05) groups (S2 Fig).

### 4. Muscle damage

**Fig 5:**
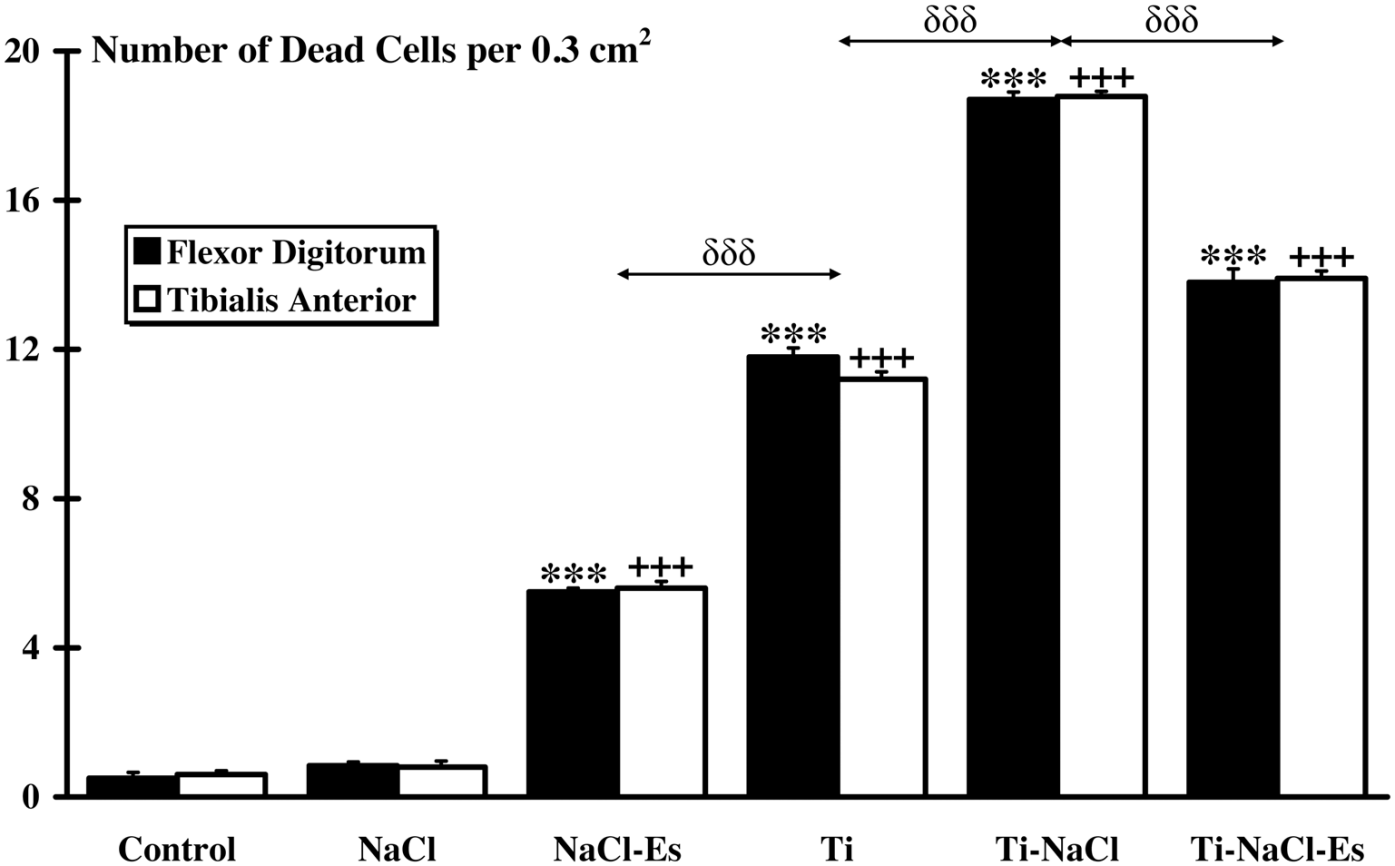
Muscle damages. Antibodies directed against activated caspase 3 are used on *flexor digitorum* and *tibialis anterior* muscles to evaluate cell death rate (number of dead cells per surface unit). No significant muscle damage is observed between muscles in each group. Furthermore, significant differences between Control and other groups are indicated by * for *flexor digitorum* and + for *tibialis anterior* (*** and +++, p<0.001). δ indicates significant intergroup differences for both muscles (δδδ, p<0.001).

No significant difference of cell death rate (expressed in number of dead cells per 0.3 cm^2^) in each group was observed between FD and TA muscles (Fig 5). No significant cell death rate was observed for both Control and NaCl groups and no significant difference was found between these two groups (Control: TA = 0.60±0.12, FD = 0.56±0.14; NaCl: TA = 0.9±0.07, FD = 0.87±0.07). Compared to Control group, cell death rate was found significantly higher in NaCl-Es group (TA = 6.1±0.18, FD = 5.95±0.10; p<0.001), Ti group (TA = 12.22±0.15, FD = 12.83±0.31; p<0.001), Ti-NaCl group (TA = 20.59±0.23, FD = 20.39±0.62; p<0.001) and Ti-NaCl-Es group (TA = 15.2±0.19, FD = 15.08±0.55; p<0.001). Moreover, higher (p<0.001) cell death was observed for all implanted groups compared to NaCl-Es group. Ti-NaCl group showed the highest (p<0.001) cell death rate compared to all other implanted groups. Furthermore, a significant difference was found between NaCl-Es and Ti-NaCl-Es (p<0.001). Finally, cell death rate did not differ between Control and Es groups (Control: 0.60±0.12 for TA, 0.56±0.14 for FD; Es: 1.07±0.12 for TA, 1.67±0.18 for FD) (S3 Fig).

## Discussion

The harmful effect of high-salt diet on blood pressure, cardiovascular, renal and bone function have been widely described [7,14]. Moreover, electrochemical features of biological tissues can be modified by high-salt diet. Chloride ions are well known to exert a corrosive effect on metallic biomaterials [15–17]. In the present *in vivo* study, the extent to which high-salt diet could influence adhesion of a Ti6Al4V implant (fixed on rat tibial crest) and surface characteristics, and could affect muscle cell survival of surrounding muscles was investigated alone and after a NMES program similar to those currently used in human care following arthroplasty. Surprisingly, high-salt diet increased implant-to-bone adhesion except when NMES program was performed. However, such a diet alone or combined with NMES induced implants deterioration. The absence of muscle damage in Es group allows us to be overcome from electrostimulation specific effects. If NMES alone do not induced muscle damage as shown in our results and in a previous study [25], its combination with high-salt diet enhanced those damage. On the other hand, our study demonstrated also that higher muscle damage was present in all implanted animals and particularly in implanted animals receiving high-salt diet.

### 1. Cell survival

For both studied muscles (TA and FD), cell death rate was similar and very low for Control, Es and NaCl groups indicating that NMES (similar than those used in human to induce rapid rehabilitation) or high-salt diet alone did not induce cell damage during a period of three weeks. However, in the present study high-salt diet combined with NMES resulted in significant muscle damage. Such process could be explained by the split of NaCl molecules into charged particles (Na^+^ and Cl^-^) that increase ionic gradient and then enhance biological tissues conductance [28]. It could be hypothesized that a NaCl-induced NMES current potentiation might impair muscular cell survival in directly stimulated muscle (FD) as in nearby muscles (like TA). To the best of our knowledge, we are the first to describe such *in vivo* interaction on muscle cells.

Compared to the other groups, cell death rate was found significantly higher when animals received a metallic implant, even for implanted animals with normal diet and without NMES. Considering Ti group, even if all precautions were taken to avoid muscle damage during surgical procedure, we cannot exclude muscular microtrauma related to FD and TA muscles separation from tibial bone. An alternate or supplemental explanation of this damage measured in Ti group could be an adverse reaction of local tissue to the metal compound. Such reaction is frequently observed in clinical cases [29]. However, regarding corrosion resistance of Ti6Al4V alloy [30] and the duration of our protocol, the last hypothesis seems unlikely. Interestingly, muscle damages were significantly higher when implanted rats received high-salt diet. This difference could not be related to surgery but to specific effect of salt interacting with the titanium implant. Many studies have demonstrated a corrosive effect of saline solution on metal [16,17] and it was reported that Na^+^ and Cl^-^ ions located in the vicinity of an implant are prone to amplify the corrosive effect on implant surface and to generate debris which in turn deteriorate biological tissues [31,32]. Moreover, in presence of Cl^-^, it was noted a fast propagation of metallic implant micro cracks and a decrease in biological pH increasing corrosive effect on metal [32]. Such process leads to inflammatory reaction leading to muscle damages [33].

Surprisingly, muscle damage rate observed in Ti-NaCl-Es group was lower compared to Ti-NaCl group but higher compared to Ti group. This result indicates a specific effect of the combination of NaCl and NMES on the implant. It is likely that the same process of degradation occurred in this last group compared to Ti-NaCl group. However, chronic stimulation is known to increase blood flow [34,35] thereby reducing accumulation of ions around the implant and finally the corrosion level. Such process might explain the lower cell death rate observed for the stimulated groups. Nevertheless, according to our results, combination of high-salt diet and NMES remains deleterious for muscle cell viability.

### 2. Implant

Despite the corrosive aspect of biological tissues, it seems that three weeks of implantation is not sufficient to induce significant modification on Ti6Al4V surface. In contrast, surface degradation was significant in Ti-NaCl group. This result suggests that high-salt diet exerts a corrosive effect on implant surface, which is in accordance with most studies reporting a corrosive effect of salt solution on metallic surface *in vitro* and *in vivo* [15,17]. Indeed, Cl^-^ are known to weaken metal-oxygen bonds forming metallic chlorides which will deteriorate materials structure [31]. Finally, degradation of implant surface was enhanced in animal receiving high-salt diet when NMES was applied, highlighting deleterious effect of such combination.

Paradoxically, the pullout test revealed that adhesion strength required for implant loosening was significantly greater for the Ti-NaCl group than the Ti and Ti-NaCl-Es groups. This is in disagreement with many studies which demonstrated a harmful effect of high-salt diet enhancing bone resorption [14,36,37]. Frings-Meuthen et al. showed that high-salt diet provokes a strengthening of tissue acidity leading to bone loss [9]. This effect might be explained by an increase in osteoclast activity in response to inflammation process induced by bone degradation related to the NaCl effects [38]. We failed to find a relevant explanation to the higher implant-to-bone adhesion in Ti-NaCl group and to the lower implant to bone adhesion in Ti-NaCl-Es group.

### 3. Conclusion

This study aimed to assess the *in vivo* effect of high-salt diet on Ti6Al4V alloy adhesion and surface characteristics and on surrounding muscle cell when associated or not to NMES program. According to our results, high-salt diet generates overall a harmful influence on cell viability and surface characteristics of the implant. This result questions the risk of such particular diet in patients with arthroplasty, especially when rehabilitation is based on NMES program. Further studies need to deepen the mechanisms involved in such process.

## Supporting Information

S1 Fig (XLS)

S2 Fig (XLS)

S3 Fig (XLS)
